# Microbiota evaluation in acute appendicitis: a preliminary study of appendix and childhood oral microbiota

**DOI:** 10.3389/froh.2025.1690433

**Published:** 2026-02-09

**Authors:** Renata Thomaz Katzenelson, Pedro Izzo, Beatriz Rissi Corrallo, Camila Lopes Crescente, Alana Cintra Alcantara, Fabíola Galbiatti de Carvalho, Leonardo Souza Marques, Emerson Tavares de Sousa, Thaís Manzano Parisotto

**Affiliations:** 1 University São Francisco—USF, Bragança Paulista, São Paulo, Brazil; 2 Federal University of Uberlândia—UFU, Uberlância, Minas Gerais, Brazil; 3 University of California, San Francisco, CA, United States

**Keywords:** intestinal microbiota, oral microbiota, acute appendicitis, dental caries, children

## Abstract

Despite the uncertainty of the sequence of events leading to appendicitis, the microbiome is presumed to play a central role in the pathogenesis. This preliminary cross-sectional study aimed to evaluate the intestinal microbes in distinct phases of acute appendicitis compared to the oral microbiota, considering the status of dental caries. Twenty children under 12 years were assigned into two groups: non-complicated appendicitis (NCA, *n* = 11) and complicated appendicitis (CA, *n* = 9). Biological material from the oral cavity (saliva) and appendix (mucosal scrapings) was submitted to microbial analysis to quantify *Bacteroidetes*, *Firmicutes*, and *Fusobacterium nucleatum* by qPCR. Data were assessed using ANOVA and Pearson's correlation (*α* = 5%). Considering CA, significant differences were found between the mouth and appendix for *Bacteroidetes*, *Firmicutes*, and *Fusobacterium nucleatum* levels (*p* < 0.05), with increased amounts in the intestinal niche. Conversely, there was no statistical difference (*p* > 0.05) regarding NCA. *Bacteroidetes* levels in the intestinal appendix significantly correlate to all of the studied bacteria in the mouth (*p* < 0.05, *r* = 0.66–0.89), while in the NCA pattern, this happened only with *Bacteroidetes*. Caries conditions were similar between the two groups. In conclusion, a relationship is suggested between the intestinal appendix and oral bacteria, in a comparable caries index, in distinct phases of acute appendicitis. The severity of the children's appendix condition seems to correlate with the magnitude of microbial changes.

## 1 Introduction

Appendicitis is a common disease among children/adults and the leading cause of acute abdominal pain. About 228 in 100,000 individuals developed this disorder in 2019, and the prevalence peak is more frequent in the second decade of life (1). Usually, boys are more commonly affected than girls (1).

In most children, the appendix is located in the right lower quadrant of the abdomen, attached to the posteromedial end of the cecum and inferior to the ileocecal junction. The tip of the appendix is around 6–10 cm long and might be located in various areas of the abdominal cavity, 60% retrocecal. In the first year of life, the appendix has a funnel shape, reducing the likelihood of obstructive processes (2). The appendix comprises a mucosa rich in lymphoid follicles interspersed with colonic epithelium. When facing a viral or bacterial process, lymphoid hyperplasia can cause luminal obstruction. Other possibilities leading to obstruction would be fecal material (fecalith), gallstones, parasites, or neoplasms (3).

The diagnosis is based on the clinical history, with an initial periumbilical pain that migrates to the right iliac fossa, associated with nausea, vomiting, low fever, and anorexia. The macroscopic appearance of appendicitis is variable, being localized or diffuse and divided into four stages (edematous, phlegmonous, gangrenous, suppurative) or just into two phases: complicated (gangrenous or perforated appendix, with or without purulent intra-abdominal fluid or abscess formation) or non-complicated (inflammation of the appendix with transmural invasion of neutrophils) (3). More specifically, in the early stages of appendicitis, the appendix may appear normal to the naked eye, with the serous portion being usual or with vascular congestion areas. Then, the appendix becomes edematous, with an external diameter exceeding 1 cm and fibrinous exudate. Later, thickness and peri-appendiceal alterations may be found, such as abscesses, gangrene, and purple/black discolorations. Perforations can occur in more severe stages (3). In a microscopic view, mucosal ulceration (localized or diffuse) might be associated with acute transmural inflammation or vascular thrombosis, leading to hemorrhagic wall necrosis (3).

Appendicitis is a multifactorial disease that could be influenced by dietary habits, environment, lifestyle, and family history, for example (4). Despite the uncertainties regarding the sequence of events leading to appendicitis, it is assumed that the microbiota has a central role in the pathogenesis (5), changing during the disease course (6–9). Among the microbial agents involved in this process, the phyla *Bacteroidetes* and *Firmicutes*, as well as the species *Fusobacterium nucleatum* (FN)*,* have already been described in the scientific literature (7–12). More importantly, the gut microbiome is dominated at the phylum level by *Firmicutes* and *Bacteroidetes* in the pediatric population (13); and the vast majority of studies on inflammatory intestinal diseases in youth have reported significant changes in these two phyla, which are the primary focus of most investigations (11–15). There is a growing interest in FN, a gram-negative, significant biofilm former anaerobic oral pathogen. Of interest, the mouth harbors the second-largest and most diverse microbial community in the body with diverse ecological niches (tongue, cheeks, gingiva, palate, teeth). A recent investigation suggested that ectopic colonization of oral *Fusobacterium* in the appendix could play a critical role in appendicitis pathogenesis (9), proliferating and persisting even with numerous weeks of broad-spectrum antibiotics before surgery (16). Still, it has been associated with cancer (17), which is an expressive disease responsible for lots of deaths among the worldwide population (18). Thus, FN, together with *Firmicutes* and *Bacteroidetes* phyla, have been selected to be investigated in the present study.

There have been only two investigations concerning the microbial composition of appendicitis in both intestinal and oral material during childhood (9, 10). However, one did not consider the severity of appendicitis (10), and the other did not consider the oral health status (9). Thus, this study aimed to evaluate the intestinal microbes in distinct phases of acute appendicitis compared to the oral microbiota, considering the caries status.

## 2 Material and methods

### 2.1 Sample

The present research has been approved by the Ethics Committee of the University São Francisco (USF) (Approval No. #44902221.0.0000.5514) and it conforms to the provisions of the Declaration of Helsinki. Seventy-two children under 12 years were preliminarily and prospectively recruited at the University's Hospital (USF), Bragança-Paulista, Brazil, with a positive consent form signed by their guardians. These children were admitted to the pediatric surgery service with a diagnosis of appendicitis between April/2020 and December/2022. Of the 72 children, twenty-seven participants were excluded because there were no available professionals to collect the biological samples (appendix scrapings and saliva), as their procedures occurred during the off-duty period of the research responsible physician, RTK. Four were excluded due to probiotics or antibiotic usage before the surgery (including in the past 30 days), seven due to Meckel diverticulum, and one due to refusal to cooperate during the oral examination and saliva collection. These patients were assigned to two categories: complicated (*n* = 11) and non-complicated (*n* = 22). To ensure a homogeneous study design, we selected eleven children with non-complicated appendicitis, matching them by sex and age with those presenting complicated cases. Unfortunately, two patients with complicated appendicitis were excluded due to the inability to recover saliva after centrifugation of the Salivette® device, as they were dehydrated. Consequently, the final sample comprised 9 patients with complicated appendicitis and 11 with non-complicated appendicitis (Supplementary Figure S1).

### 2.2 Diagnosis of appendicitis

The diagnosis of appendicitis was made through a combination of clinical history, physical examination, and blood test results, based on the Alvarado score (19). It is a clinical scoring tool designed to assist in diagnosing acute appendicitis and utilizes a 10-point scale derived from eight clinical signs and laboratory results, where higher scores indicate a greater likelihood of having appendicitis. Patients' characteristics, including weight, height, age, medical history, lifestyle information, blood test results, imaging test results, and antibiotic use, were obtained from medical records.

The complicated group of appendicitis included the gangrenous and perforated types, whereas the non-complicated included the edematous and phlegmonous. The edematous stage represents the initial phase of appendicitis, characterized by swelling resulting from fluid accumulation within the tissue, which is a typical early manifestation of inflammation. The phlegmonous stage follows, marked by the progression of inflammation extending beyond the appendix to the surrounding soft tissues. The most severe stages are gangrenous and perforated appendicitis. The gangrenous stage signifies tissue necrosis resulting from a compromised blood supply or severe infection. The perforated stage indicates the formation of a rupture or perforation in the appendix wall, allowing the release of infectious material into the abdominal cavity, which significantly increases the risk of severe complications.

### 2.3 Collection of intestinal appendix material

A doctor (RTK) specialist in Pediatric Surgery scraped the inflamed mucosa of the appendix using a sterile scalpel blade during the surgical intervention. The scraps were stored in microcentrifuge tubes and immediately held at −20 °C until the surgery was completed. Then, they were transferred to −80 °C until the microbiological analysis.

### 2.4 Assessment of oral health status and saliva collection

Caries' diagnosis was made by visual inspection at the hospital. A mirror and a ball-ended probe were used following the WHO (World Health Organization) criteria (20). Two calibrated dentists (Kappa intra/interexaminer: 0.80/0.86) carried out the exams. To collect a saliva sample, the Salivette® (Sarstedt, Germany) device was opened, the swab was removed, and placed in the mouth. After 60 s the swab was returned to the tube for subsequent centrifugation (2 min, 1,000 g) and freezing at −80 °C until the analyses.

### 2.5 DNA extraction and real-time PCR

A specific DNA extraction kit (MasterPure™ Complete DNA Purification Kit, Cat.#MC85200, Holliston-MA/USA) was used to extract DNA from saliva and appendix samples. Real-time PCR assays were performed on the 7,300 Real-Time System (Applied Biosystems, Foster City-CA/USA). The primer sequences used in this study have already been reported in the scientific literature. For *Firmicutes*, the forward primer was 5′-GGAGYATGTGGTTTAATTCGAAGCA-3′ and the reverse primer was 5′-AGCTGACGACAACCATGCAC-3′ (21). For *Bacteroidetes*, the forward primer was 5′-GGARCATGTGGTTTAATTCGATGAT-3′ and the reverse primer was 5′-AGCTGACGACAACCATGCAG-3′ (21). For FN, the forward primer was 5′-CAACCATTACTTTAACTCTACCATGTTCA-3′ and the reverse primer was 5′-GTTGACTTTACAGAAGGAGATTATGTAAAAATC-3′ (22). Primers were designed based on the 16S rRNA gene. However, *Firmicutes* and *Bacteroidetes* primers were designed based on conserved regions of the 16S rRNA gene found in a wide range of gut and oral bacterial species from these two phyla, as listed in GenBank. All primers were assessed for specificity using the BLAST tool (Basic Local Alignment Search Tool—http://www.ncbi.nlm.nih.gov/blast/) and were confirmed to be highly specific for their target sequences. A total of 1.5 μL of DNA extracted from the samples was used for the assay together with five μL of SYBR Green Power up (Thermo Fisher Scientific, Carlsbad-CA/USA), 2.9 μL of water, and 0.3 μL of each primer (Forward/Reverse). The real-time PCR programs for detecting *Bacteroidetes*, *Firmicutes*, and FN included 2 min at 50 °C, 10 min at 95 °C, 40 cycles of 15 s at 95 °C, and 1 min at 60 °C.

*Clostridium perfringens* (ATCC 13124), *Bacteroides fragilis* (ATCC 25285), and a clinical sample of FN (previously identified in the Laboratory of Clinical Microbiology of University São Francisco) were employed as positive controls for the *Firmicutes*, *Bacteroidetes* phyla, and FN*,* respectively. DNA from these control strains was diluted in a series of ten-fold steps (ranging from 10^1^ to 10^5^ ng/μL across five concentrations) to create standard curves used for quantifying the absolute amount of target DNA in the test samples. Specifically, the Sequence Detection Software (version 1.3.1, Applied Biosystems, Foster City, CA, USA) monitored the amplification of target sequences in both the standard dilutions and the unknown samples. Based on the amplification data from the dilution series, the standard curve was constructed. The software then used this curve to estimate the absolute concentration of the target DNA present in each test sample.

The critical threshold cycle had detectable fluorescence above the background (threshold:0.200). All qPCR was performed in duplicate. Bacterial concentration was expressed in ng DNA/μL, considering that the samples were standardized based on the DNA concentration detected in the Biodrop equipment (Biochrom, Holliston, MA, USA).

### 2.6 Statistical analysis

Data were analyzed using SPSS for Windows, version 21.0 (SPSS Inc., Chicago, IL, USA), with a significance level of *α* = 5%. The normality of the sample distribution was assessed using the Shapiro–Wilk test, which indicated that the variables did not follow a Gaussian distribution. Therefore, lognormal (*Bacteroidetes* and *Firmicutes*) and square-root (FN) transformations were applied to meet ANOVA assumptions. A two-way repeated-measures analysis of variance was conducted to evaluate the interaction between anatomical location (oral cavity vs. intestinal appendix) and appendicitis type (non-complicated vs. complicated). The equality of multiple variance–covariance matrices was confirmed using Box's M test, with a significance level of 0.001. The simple effects test was conducted as a single-step pairwise comparison between children with complicated and non-complicated appendicitis, within the oral cavity and appendix, considering *Bacteroidetes, Firmicutes*, and FN. In addition, alpha (*α*), beta (*β*), and partial eta squared (*η*_p_^2^) represent, respectively, the type I error, the type II error, and the effect size.

## 3 Results

### 3.1 Study subjects/oral health status

This preliminary study examined children admitted with abdominal pain to the Hospital of the University São Francisco for 32 months (2020–2022). The sample characteristics are displayed in Table 1.

**Table 1:** Sample characteristics according to the stage of appendicitis: complicated and non-complicated.

	**Non-complicated appendicitis (NCA** **=** **11)**	**Complicated appendicitis (CA** **=** **9)**
	Median (IQR)	
Age (years)	8.18 (2.4)	7.00 (3.6)
BMI (Kg/m^2^)	15.74 (5.9)	19.17 (4.9)
Alvarado Score	5.00 (3.0)	8.00 (2.0)
White blood cell count	10,100.0 (8,700.0)	15,100.0 (5,900.0)
Length of hospital stay	3.0 (1.0)	6.0 (2.0)
Total Caries Index	5.00 (6.00)	4.00 (6.00)
	n (%)	
Girls	5 (45)	4 (45)
Boys	6 (55)	5 (55)
Presence of dental biofilm	11 (100)	8 (90)
Caries-free	3 (27)	3 (33)

IQR, interquartile range; %, percentage; Total caries index, dmft (decayed missing or filled primary teeth) + DMFT (decayed missing or filled permanent teeth).

The age range was reasonably equivalent between the NCA and the CA groups. BMI, Alvarado scores, white blood cell count, and length of hospital stay were superior in children with complicated appendicitis. The two studied groups showed similar results in the presence of dental biofilm on the maxillary incisors, the percentage of children free of caries, and the total caries index (Table 1). Regarding the rate of each component of the total caries index, the fillings accounted for the highest percentage in both groups (CA 65%; NCA 40%), followed by decayed (CA 16%; NCA 20.5%), missing as a result of caries (CA 13%; NCA 20.5%), and filled with decay (CA 6%; NCA 19%). Considering the index in primary (dmft) and permanent (DMFT) teeth separately, the medians were 4 and 0 for the NC group and 2 and 0 for the complicated group.

### 3.2 Microbiological analysis

In Table 2, overall, the appendicitis severity pattern may promote significant disturbances in the bacterial ecosystem of the oral cavity and intestinal appendix. This can be proved by the strong and significant statistical differences between the oral cavity and appendix when appendicitis is categorized as complicated regarding the levels of *Bacteroidetes* [*α* = 0.004 (*β* − 1 = 0.879) *η*_p_^2^ = 0.410], *Firmicutes* [*α* <0.001 (*β* − 1 = 0.999) *η*_p_^2^ = 0.581], and FN [*α* =0.012 (*β* − 1 = 0.747) *η*_p_^2^ = 0.276] (Simple effects for Anatomic location and Appendicitis pattern). Regarding the same microbes, statistical differences were not found between the mouth and appendix for non-complicated appendicitis. Still, Table 2 shows a significant interaction between anatomical location and appendicitis pattern for *Firmicutes* only. At the same time, an *α* value at the margin of statistical non-significance was achieved for *Bacteroidetes* (*p* = 0.056).

**Table 2:** Interaction between anatomic location (oral cavity × intestinal appendix) and appendicitis pattern (non-complicated × complicated): a Two-way mixed model.

Appendicitis Pattern	Bacteroidetes		Firmicutes		Fusobacterium nucleatum	
	Oral cavity	Intestinal appendix	Oral cavity	Intestinal appendix	Oral cavity	Intestinal appendix
Non-complicated	0.40 (3.42) Aa	0.18 (0.18) Aa	2.70 (5.99) Aa	1.28 (1.85) Aa	0.14 (0.53) Aa	0.13 (0.26) Aa
Complicated	0.42 (3.18) Aa	3.19 (10.37) Bb	0.86 (5.17) Ba	24.19 (20.90) Bb	0.16 (0.28) Aa	1.00 (1.80) Ab
Two-way Mixed Model	*p*-value (Power)	*η*_p_^2^	*p*-value (Power)	η_p_^2^	*p*-value (Power)	*η*_p_^2^
Anatomic Location	0.044* (0. 536)	0.229	0.010* (0.767)	0.285	0.024* (0.641)	0.229
Appendicitis Pattern	0.040* (0.554)	0.237	0.025* (0.637)	0.228	0.183 (0.259)	0.087
Anatomic Location * Appendicitis Pattern	0.056 (0.489)	0.209	<0.001* (0.993)	0.517	0.159 (0.286)	0.097

Data were expressed in ng DNA/μL and plotted as median and interquartile range (due to non-Gaussian distribution). The lognormal (*Bacteroidetes* and *Firmicutes*) and square root (FN) transformations were used to fulfill the ANOVA premises. Asterisks indicate statistical significance (*p* < 0.05). *Different capital letters indicate statistically significant differences in each column (non-complicated x complicated). Distinct lower letters indicate statistically significant differences between lines (oral cavity x intestine). To improve visualization, pointed lines reinforce significant comparison in each column (non-complicated vs. complicated), and grey highlights reinforce significant comparison between lines (oral cavity vs. intestine). *η*_p_^2^: Partial eta squared.

Simple effects for *Bacteroidetes:* Appendicitis Pattern  ×  *Bacteroidetes* levels in the Oral cavity [*α* = 0.048 (*β* − 1 = 0.522) *η*_p_^2^ = 0.223] and Intestine [*α* = 0.025 (*β* − 1 = 0.641) *η*_p_^2^ = 0.276]. Simple effects for Anatomic location in non-complicated [*α* = 0.856 (*β* − 1 = 0.053) *η*_p_^2^ = 0.002] and complicated appendicitis [*α* = 0.004 (*β* − 1 = 0.879) *η*_p_^2^ = 0.410].

Simple effects for *Firmicutes:* Appendicitis Pattern  ×  *Firmicutes* levels in the Oral cavity [*α* = 0.031 (*β* − 1 = 0.599) *η*_p_^2^ = 0.212] and Intestine [*α* < 0.001 (*β* − 1 = 0.975) *η*_p_^2^ = 0.460]. Simple effects for Anatomic location in non-complicated [*α* = 0.218 (*β* − 1 = 0.228) *η*_p_^2^ = 0.075] and complicated appendicitis [*α* <0.001 (*β* − 1 = 0.999) *η*_p_^2^ = 0.581].

Simple effects for *Fusobacterium nucleatum*: Appendicitis Pattern  ×  *Fusobacterium nucleatum* levels in the Oral cavity [*α* = 0.868 (*β* − 1 = 0.053) *η*_p_^2^ = 0.001] and Intestine [*α* = 151 (*β* − 1 = 0.296) *η*_p_^2^ = 0.100]. Simple effects for Anatomic location in non-complicated [*α* = 0.497 (*β* − = 0.101) *η*_p_^2^ = 0.023] and complicated appendicitis [*α* =0.012 (*β* − 1 = 0.747) *η*_p_^2^ = 0.276].

95% Confidence Intervals are available in Supplementary Table S1.

**Figure 1:**
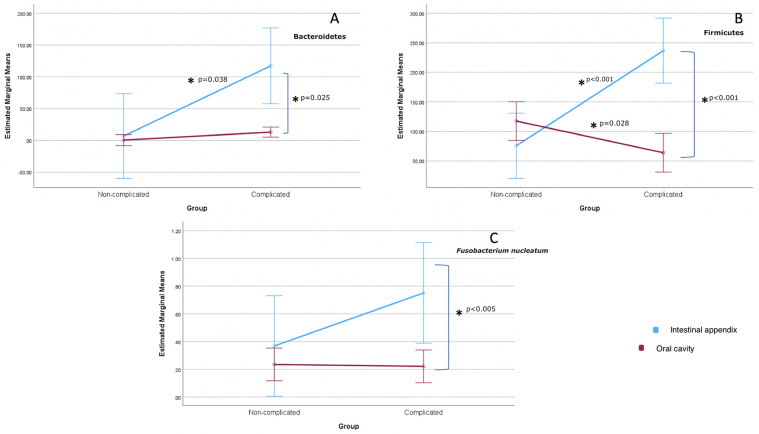
Profile plots of anatomic location (mouth × intestine) against the appendicitis pattern (non-complicated × complicated). The blue and pink lines represent the intestine and oral cavity, respectively. Each letter represents a distinct type of bacteria: *Bacteroidetes* **(A)**, *Firmicutes* **(B)**, and *Fusobacterium nucleatum* **(C)** A single asterisk denotes a significant *p*-value (<0.05) by ANOVA Two-way Mixed Model.

Figure 1, corroborating Table 2, demonstrates that the level of *Bacteroidetes* is expressively higher in the intestinal appendix of children with complicated appendicitis, showing a statistical difference compared to the oral cavity (*p* < 0.05—Figure 1A). The level of *Firmicutes* is also higher in the appendix of complicated appendicitis compared with non-complicated appendicitis (*p* < 0.05). The concentration of this phylum tends to be lower in the oral cavity (Figure 1B) of those with a complicated pattern compared to the non-complicated ones (*p* < 0.05). FN amounts behave similarly to *Bacteroidetes* in the oral cavity and intestinal appendix, but the positive inclination in the appendix was non-significant (*p* > 0.05—Figure 1C).

Table 3 shows the Pearson Correlation Matrix between *Bacteroidetes*, *Firmicutes*, and FN. Among the three proposed correlation models, Model 3, which includes complicated appendicitis cases, exhibited more positive correlations considering *Bacteroidetes* in the appendix vs. *Firmicutes*, FN*,* and *Bacteroidetes* in the mouth (*p* < 0.05, *r* = 0.662–0.893). In contrast, Model 2, representing non-complicated appendicitis, exhibited only one strong correlation between *Bacteroidetes* in the appendix and in the oral cavity. The same pattern was observed in Model 1. Notably, this specific correlation was consistently present across all three models.

**Table 3:** Pearson correlation matrix between Bacteroidetes, Firmicutes, and Fusobacterium nucleatum.

Variables	Model 1: overall		Model 2: appendicitis non-complicated		Model 3: appendicitis complicated	
	Pearson correlation (95% CI)	*p*-value (2-tailed)	Pearson correlation (95% CI)	*p*-value (2-tailed)	Pearson correlation (95% CI)	*p*-value (2-tailed)
*Bacteroidetes* Appendix vs. Bacterioidetes Oral Cavity	0.687 (0.36–0.86)	<0.001	0.948 (0.79–0.99)	<0.001	0.893 (0.61–0.97)	<0.001*
*Bacteroidetes* Appendix vs. *Firmicutes* Oral Cavity	–	–	–	–	0.662 (0.07–0.90)	0.026*
*Bacteroidetes Fusobacterium nucleatum* Oral Cavity	–	–	–	–	0.880 (0.56–0.97)	<0.001*

Non-significant correlations were suppressed. Pearson's estimation was based on Fisher's t-to-z transformation with bias adjustment. Asterisks indicate statistical significance.

## 4 Discussion

To the best of our knowledge, the present preliminary study shows, for the first time, a relationship between the intestinal appendix and oral microbiota in the distinct phases of acute appendicitis in children with a similar dental caries index. More specifically, while in the mild degree, no significant differences were found between the mouth and appendix regarding two of the most predominant phyla, *Bacteroidetes* and *Firmicutes*, and the species FN, the opposite occurred in the severe condition (Table 2, Figure 1). This could probably be explained by the microbial dysbiosis happening in the pathophysiology of the diseases, as shown in the scientific literature (23, 24). Notably, recent research assessing acute appendicitis in childhood proved that gut microbial components differ significantly in pediatric patients with acute appendicitis compared to healthy ones (9) and indicated that the divergent abundance of specific microbes is connected to the production of appendicitis inflammatory markers (25).

Our research supposes that the higher the severity of the disorder, the more the imbalance in the microbiota, especially in the appendix scraps (Table 2/Figure 1). This aligns with the alterations that occur during disease progression (6, 10, 26, 27), since the early stages of appendicitis, until the peri-appendiceal alterations (abscesses, gangrene, and purple/black discolorations) and perforations (3) in the most severe stage.

It is curious to highlight that independent of the degree of appendicitis severity, a positive and significant correlation was found between *Bacteroidetes* levels in the appendix and the oral cavity (Table 3). This suggests that the oral cavity may reflect, to some extent, the microbial conditions of the gut. This is an intriguing finding, as saliva collection is easier and minimally invasive, offering a practical approach for microbiota monitoring. In this respect, a recent study highlighted that precise biomarkers discerning complexes from uncomplicated appendicitis in children are lacking and are of prime importance, as both stages usually require distinct treatment strategies (28). The authors also suggested rectal swabs before surgery to distinguish the simple from the complex appendicitis (28). Considering that minimal interventions are always welcome for children, the possibility of using oral swabs could be raised. This observation is compelling considering the anatomical and physiological continuity between the oral cavity and the gastrointestinal tract.

In complicated appendicitis, *Bacteroidetes* levels in the intestinal appendix significantly correlate with all the studied bacteria in the mouth (*Bacteroidetes*, *Firmicutes*, and FN*–*Table 3). At the same time, in the non-complicated pattern, this has happened only with *Bacteroidetes*. It could be supposed that great changes are happening in bacteria from this phylum in the gut appendix, possibly the classical *Bacteroides fragilis*, which could have led to a significant difference with the bacteria from the oral cavity. Our findings align with data supporting that the mouth seems to be a reservoir for microbes, resulting in inflammatory systemic reactions (9, 10, 21, 24).

Similar to *Bacteroidetes*, the level of *Firmicutes* is also higher in the appendix in the complicated cases compared to non-complicated ones (Table 2). Also, the concentration of this phylum tends to be lower in the oral cavity (Figure 1B) in a complex pattern. A plausible reason may be the microbial imbalance in the mouth, where, according to the characteristics of this niche, microbes from other phyla, which were not investigated in the present study, could have been favored.

FN behaves similarly to *Bacteroidetes* (Figure 1) and tends to increase with the severity of the disease, not reaching statistical significance, probably due to the sample variability, as demonstrated by the interquartile range (Table 2). A previous study has pointed to considerable interindividual variability in the microbial composition of the appendix samples (26). Authors have also shown that the prevalence of *Fusobacterium* (genus) could be connected to the appendix inflammation severity (26), including a recently published paper (9). Papers also supported the idea that acute appendicitis in children was linked to an abundance of *Fusobacteria* (7, 16), including FN (16). A current published investigation also suggested that *Fusobacterium* spp. could act as an indicator for the appendicitis severity in childhood (8). Notably, the two published studies investigating the microbial composition of pediatric appendicitis in intestinal and oral samples suggested a possible association between FN and periodontal or endodontic diseases, although this was not specifically assessed in their samples (9, 10). Both studies acknowledged that bacterial migration from the oral cavity, through the stomach, to the appendix is plausible. One study even evaluated the postprandial viability of bacteria, reporting that FN exhibited consistent growth at pH 6 and 5 throughout the entire incubation period of 0–240 min, and remained cultivable after 30 min of exposure to a nutrient broth at pH 4 (10).

In the intestinal appendix, significantly higher amounts of *Bacteroidetes*, *Firmicutes*, and FN were found in the severe pattern compared to the mild one (Table 2). This happened because the more problematic the situation, the more microbes are expected, corroborating previous studies (6, 26). On the other hand, when the mouth levels were considered, significant differences were observed only with *Firmicutes* levels (Table 2), with lower amounts in the severe pattern. As this is the most prevalent phylum in the oral cavity, changes in bacteria from this phylum could be responsible for dysbiosis.

The present investigation found a similar total caries index (Table 1). Fillings were the most prevalent lesion pattern in NCA and CA groups, followed by cavitation, missing teeth due to caries, and teeth filled with decay. Primary teeth were more affected by caries lesions in both groups compared with the permanent ones. This finding was expected, as caries severity increases with age. In our study, we included children aged 7–8 years who had primary teeth in their mouths for several years and were experiencing the newly erupted permanent teeth. The high number of filled teeth might indicate the children had dental treatment assessments, probably because of school enrollment, as a dentist's notification letter regarding oral health conditions is required in the municipality of Bragança. Assessing oral health is essential for understanding the distinct ecological niches available for microbial colonization. This is because dental surfaces affected by carious lesions favor microbial establishment, acting as retentive ecological niches, since an increased roughness (due to initial carious lesion demineralization) or different textures (fillings vs. enamel) until frank cavities.

Despite the novel findings, the present investigation also has limitations. We have used qPCR, and studies with sequencing methodology could provide additional information, mainly including other phyla. Additionally, identifying specific species, including cariogenic ones such as *Streptococcus mutans*, would be valuable for supporting the hypothesis of bacterial translocation from the oral cavity to the gut. Due to the preliminary character of the present study, the limited number of appendectomy cases enables a moderate to high power in the analysis but precludes the largest. Further prospective studies should be stimulated to overcome these gaps, including more children with different oral health conditions.

Considering that acute appendicitis is common in surgical emergencies and is associated with severe complications and high morbidities (29, 30), the choice of the best treatment strategy is essential. In this respect, microbial information could be helpful, especially in simple cases where empirical antibiotic therapy could be discussed, targeting assertive implementation to prevent resistance (31). Intriguingly, current research proposed that antibiotic usage during childhood might increase appendicitis risk (32). It is well known that these medications promote dysbiosis and that the gut harbors the largest microbial population in the human body. The microbial balance is critical for healthy conditions, particularly in childhood, a crucial period with rapid growth and development, where actions could shape their future. The linkage between oral bacteria and systemic disturbances reinforces the importance of common risk approaches regarding oral and general diseases. Furthermore, simple, accessible diagnosis and microbial indicators in children's saliva might improve appendicitis identification and may help with antibiotic indication when appropriate.

In conclusion, the present preliminary study suggests a relationship between the gut appendix microbes in the distinct phases of acute appendicitis and oral bacteria in similar caries status. Moreover, *Bacteroidetes*, *Firmicutes*, and FN seemed to play role in acute appendicitis pathogenesis, particularly in complex cases.

## Data Availability

The raw data supporting the conclusions of this article will be made available by the authors, without undue reservation.
