# Supplementary figures and images for: Microbiota evaluation in acute appendicitis: a preliminary study of appendix and childhood oral microbiota
Source: Front Oral Health. 2026 Feb 9;6:1690433. doi: 10.3389/froh.2025.1690433 (PMC12926399; doi:10.3389/froh.2025.1690433)

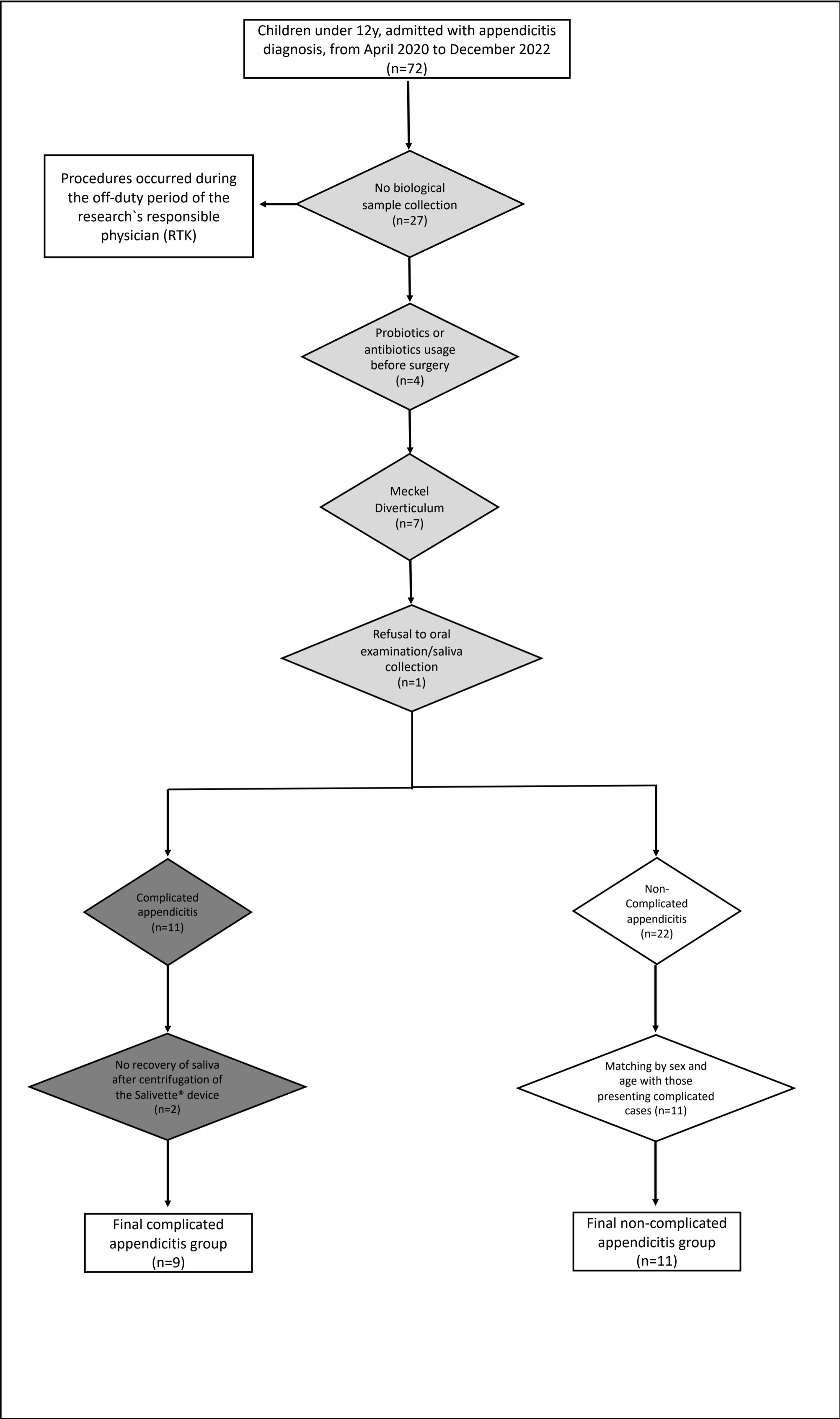

**Supplementary Figure 1.** Flow chart of the sample selection.

Supplement: Supplementary file 1 [file Datasheet1.pdf]
