# Supplementary material for: Microbiota evaluation in acute appendicitis: a preliminary study of appendix and childhood oral microbiota
Source: Front Oral Health. 2026 Feb 9;6:1690433. doi: 10.3389/froh.2025.1690433 (PMC12926399; doi:10.3389/froh.2025.1690433)
Supplement: Supplementary file 2 [file Table1.docx]

| **Bacterial Phylum/specie** | **Anatomical location** | **Appendicitis type** | **Median** | **95% CI Lower** | **95% CI Upper** |
| --- | --- | --- | --- | --- | --- |
| *Bacteroidetes* | Intestinal appendix | Complicated | 3.19 | 0.555484189 | 13.89390535 |
| *Bacteroidetes* | Intestinal appendix | Non-complicated | 0.18 | 0.133590091 | 0.303417107 |
| *Bacteroidetes* | Oral cavity | Complicated | 0.42 | 0.040557613 | 3.003601119 |
| *Bacteroidetes* | Oral cavity | Non-complicated | 0.4 | 0.044206404 | 3.383278668 |
|  |  |  |  |  |  |
| *Firmicutes* | Intestinal appendix | Complicated | 24.19 | 12.34226778 | 30.67914851 |
| *Firmicutes* | Intestinal appendix | Non-complicated | 1.28 | 0.031093078 | 23.82074268 |
| *Firmicutes* | Oral cavity | Complicated | 0.86 | 0.035792345 | 4.535249662 |
| *Firmicutes* | Oral cavity | Non-complicated | 2.7 | 0.881507548 | 8.333967152 |
|  |  |  |  |  |  |
| *Fusobacterium nucleatum* | Intestinal appendix | Complicated | 1 | 0.427287548 | 2.198227014 |
| *Fusobacterium nucleatum* | Intestinal appendix | Non-complicated | 0.13 | 0.034570613 | 0.29075509 |
| *Fusobacterium nucleatum* | Oral cavity | Complicated | 0.16 | 0.076498212 | 0.366233998 |
| *Fusobacterium nucleatum* | Oral cavity | Non-complicated | 0.14 | 0.051545278 | 0.667636328 |

**Supplementary Table 1.** Description of the 95% Confidence Intervals.
